# A Mathematical Model of Immune Activation with a Unified Self-Nonself Concept

**DOI:** 10.3389/fimmu.2013.00474

**Published:** 2013-12-26

**Authors:** Sahamoddin Khailaie, Fariba Bahrami, Mahyar Janahmadi, Pedro Milanez-Almeida, Jochen Huehn, Michael Meyer-Hermann

**Affiliations:** ^1^Department of Systems Immunology, Helmholtz Centre for Infection Research, Braunschweig, Germany; ^2^CIPCE, School of Electrical and Computer Engineering, College of Engineering, University of Tehran, Tehran, Iran; ^3^Neuroscience Research Centre and Department of Physiology, Faculty of Medicine, Shahid Beheshti University of Medical Sciences, Tehran, Iran; ^4^Department of Experimental Immunology, Helmholtz Centre for Infection Research, Braunschweig, Germany; ^5^Bio Centre for Life Science, Technische Universität Braunschweig, Braunschweig, Germany

**Keywords:** immune activation, autoimmunity, autoreactive T cells, regulatory T cells, central tolerance, peripheral tolerance

## Abstract

The adaptive immune system reacts against pathogenic nonself, whereas it normally remains tolerant to self. The initiation of an immune response requires a critical antigen(Ag)-stimulation and a critical number of Ag-specific T cells. Autoreactive T cells are not completely deleted by thymic selection and partially present in the periphery of healthy individuals that respond in certain physiological conditions. A number of experimental and theoretical models are based on the concept that structural differences discriminate self from nonself. In this article, we establish a mathematical model for immune activation in which self and nonself are not distinguished. The model considers the dynamic interplay of conventional T cells, regulatory T cells (Tregs), and IL-2 molecules and shows that the renewal rate ratio of resting Tregs to naïve T cells as well as the proliferation rate of activated T cells determine the probability of immune stimulation. The actual initiation of an immune response, however, relies on the absolute renewal rate of naïve T cells. This result suggests that thymic selection reduces the probability of autoimmunity by increasing the Ag-stimulation threshold of self reaction which is established by selection of a low number of low-avidity autoreactive T cells balanced with a proper number of Tregs. The stability analysis of the ordinary differential equation model reveals three different possible immune reactions depending on critical levels of Ag-stimulation: a subcritical stimulation, a threshold stimulation inducing a proper immune response, and an overcritical stimulation leading to chronic co-existence of Ag and immune activity. The model exhibits oscillatory solutions in the case of persistent but moderate Ag-stimulation, while the system returns to the homeostatic state upon Ag clearance. In this unifying concept, self and nonself appear as a result of shifted Ag-stimulation thresholds which delineate these three regimes of immune activation.

## Introduction

The immune system is continuously exposed to a wide variety of disturbances. Such disturbances are recognized by T cells via antigen presentation. Antigen presentation is a process in which antigen presenting cells (APC) capture the antigens, break them into small peptides, couple them with MHC molecules, and present them on the cell surface, thus enabling their recognition by T cells (1–3). The majority of disturbances that the immune system deals with are pathogenic nonself-antigens. Since the APCs break down the nonself-antigens into smaller peptides and present them on their surface, presented peptide of nonself might have overlaps with self-peptides (4, 5).

**Figure 1:**
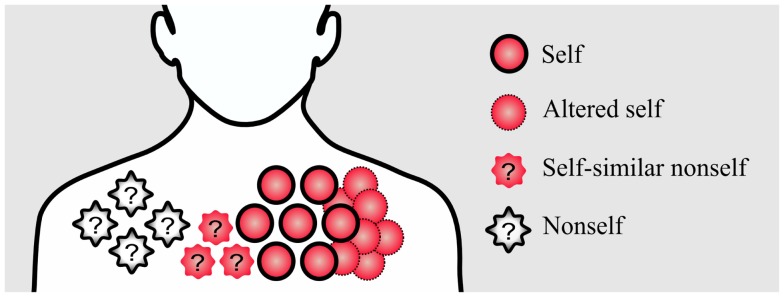
**Conceptual figure of different disturbances in the immune system**. Pathogenic nonself-disturbances are recognized and attenuated by nonself-specific T cells. However, recognition and attenuation of altered self and self-similar nonself-disturbances is challenging for the immune system due to the existence of self-tolerance mechanisms; without self-specific immune cells, the immune system is not able to initiate an immune response against these disturbances.

In addition, rapidly evolving nonself pathogens, such as Hepatitis C virus, might acquire similarities to self-antigens (6). Apart from nonself, altered self such as cancer cells is also a disturbance that has to be recognized by the immune system. Therefore, an ideal immune system has to find a solution for dealing with nonself, self-similar nonself, and self-disturbances (Figure 1).

As a general solution, the immune system generates T cell clones with random specificities that could potentially recognize any peptides, including self-peptides. The classical idea that the T cell repertoire has to be self-tolerant and T cells should not react to self-peptides, assumes that self-reactive T cells should be eliminated. This assumption is partially true, as T cell clones which fully recognize self-peptides in the thymus undergo clonal deletion, in the so-called negative selection process (7, 8).

The self-tolerance resulting from negative selection is called central tolerance. A stringent central tolerance induction and deletion of all autoreactive T cells is believed to create holes in the specificity space of the T cell repertoire (9, 10) by prohibiting immune responses against self-similar nonself and altered self. Hence, a too stringent central tolerance does not seem beneficial. In line with this idea, there is evidence that negative selection only partially deletes autoreactive T cells because availability of self-peptides required for negative selection in the thymus is limited and T cells spend only a limited time in the process of thymic selection (11–13). Autoreactive T cells escaping negative selection have been shown to be involved in autoimmunity (14). They normally exist in healthy individuals and are quiescent in steady state conditions in the presence of their cognate self-antigen (15).

Escaped autoreactive T cells are under the control of peripheral tolerance. A prominent mechanism of peripheral tolerance among others [reviewed in Ref. (16)] is induced by CD4^+^ Foxp3^+^ regulatory T cells (Tregs) (17). The majority of these cells, known as natural Tregs, are hypothesized to be selected from autoreactive T cells in thymus (18, 19). The main role of Tregs is the regulation of the immune response by suppression of the effector functions of conventional T cells (Tconv).

Despite the necessity of suppression by Tregs for avoiding autoimmunity (20, 21), production of too large numbers of Tregs in the thymus might prevent beneficial effector responses. Therefore, a too stringent peripheral tolerance induction by selection of large numbers of Tregs in the thymus does not seem favorable.

In view of this background, how does the immune system balance the tolerance mechanisms in order to ensure immune responses to any kind of disturbances including self-disturbances, yet staying tolerant to self in healthy homeostasis? Here, we address this question by using a mathematical model of immune activation that relies on identical components for self and nonself, i.e., using the same set of ordinary differential equations. The model considers the thymic production of Tregs and Tconvs as well as the dynamic interplay between Tregs, Tconvs, and IL-2 molecules in the presence of antigen(Ag)-stimulation in the periphery. The model is exploited to reveal the parametric regime of the immune system in which an immune response against self is restricted, but not impossible.

The interplay between Tregs and Tconvs during immune responses is a topic of extensive mathematical modeling (22–28). León and co-workers (22) proposed a series of models for studying immune tolerance by considering APCs, Tconvs, and Tregs. Their models rely on the assumption that regulatory interaction between Tregs and Tconvs takes place only in simultaneous conjugation with an APC. As a result of this assumption, efficient suppression of Tconvs requires a minimum population of Tregs per APC (29). As an extension, a crossregulation model is proposed by Carneiro and co-workers (26) in an attempt to incorporate Tregs in a coherent theory of the immune system. According to their model that shows a bistable behavior, immunity to a given Ag arises as competitive exclusion of Tregs by the expansion of Tconvs and tolerance results from limited APC availability or above threshold Treg numbers. Since the interactions between Tregs and Tconvs is assumed to depend on the density of the APCs, increasing the APC availability decreases the simultaneous conjugate formation of Tregs and Tconvs with the same APCs and hence, it is sufficient to break the immune tolerance.

An alternative concept was brought forward in a model proposed by Carneiro and co-workers (23) that assumes APC-independent interactions between Tconvs and Tregs for immune suppression which will be also used in our model. A direct interaction of Tconvs and Tregs was identified by experiments (30). The authors concluded that efficient immune suppression still requires a minimum population of Tregs regardless of the number of APCs.

In contrast to the aforementioned studies, we do not consider the conjugate formation of Tregs and Tconvs with APCs and therefore, there is not a competition between these cells for Ag. Instead, the role of APCs is indirectly captured by an Ag-stimulation factor which is the activation rate of naïve T cells and resting Tregs with identical Ag-specificity by APCs bearing their cognate Ag. In addition, we explicitly consider the dependency of Tregs on Tconvs through the growth factor IL-2.

Burroughs and co-workers (24) investigated Treg-induced inhibition of cytokine secretion by effector T cells. By assuming that Tregs are activated by self Ag and locally maintained by nonlinear competition for tissue-derived cytokines that are solely utilized by Tregs, the authors analyzed the role of local active Treg population size in the balance between suppressor and effector responses. Stimulation of Tregs and Tconvs is described by independent parameters. In contrast to their model, thymic output maintains the homeostatic population of Tregs in our model. Another essential difference is that Ag-stimulation of Tregs and Tconvs is described with a unified self-nonself concept and Tregs are assumed to also respond to nonself Ag-stimulation (31).

Parametric steady state analysis of the model provides insights about the physiological range of model parameters, and determines the overall conditions under which immune responses against self are possible. Furthermore, the impact of model parameters on the requirements for the initiation of immune reactions against self is analyzed. The model proposes that disturbed homeostatic balance between autoreactive T cells and Tregs increases the susceptibility to autoimmunity or cancer.

## Results

**Figure 2:**
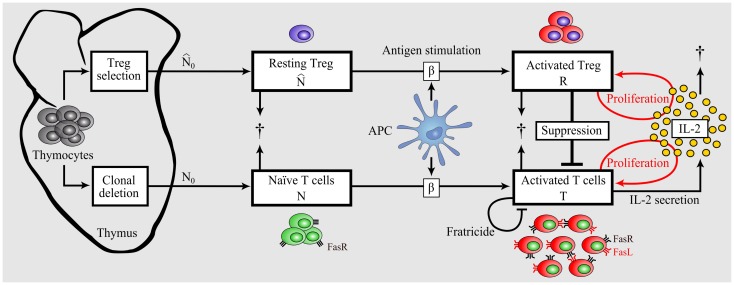
**Model of dynamic interplay between conventional T cells and regulatory T cells**. Nonself-specific as well as some self-specific thymocytes that survived negative selection and were not selected as Tregs enter the periphery as naïve T cells. A part of detected autoreactive thymocytes differentiate into Tregs in the thymus and reside in the periphery in resting state. Upon Ag-stimulation by APCs, naïve T cells and resting Tregs become activated. In contrast to activated T cells, activated Tregs do not secrete IL-2, but both activated populations proliferate in dependence on the presence of IL-2 (46). Activated Tregs suppress activated T cells in a cell-contact- dependent and cytokine-driven manner. Activated T cells undergo Fas- induced apoptosis by interacting with each other (fratricide). In contrast, Tregs are resistant to Fas-induced apoptosis (68).

The mathematical model is constructed starting from a simple model of the immune response including essential components only. Then, additional complexity is incrementally added to the model to a degree allowing for validation and analysis of tolerance versus immunity. The scheme of the complete model is depicted in Figure 2. The model is conceptually independent of the self/nonself nature of the immune response, and differences of the immune responses against self versus nonself are reflected in different parameter values of the same model.

### An immune response requires sufficient division and IL-2 secretion rate of activated T cells

Immune responses arise from massive proliferation of activated T cells and their subsequent effector function. Our simplest model attempts to capture the dynamic characteristics of an activated T cell population (*T*):

(1) $$\left\{\begin{array}{c} \frac{dT}{dt}=aIT-bT\;\\ \frac{dI}{dt}=dT-eIT-fI\;\\ \; \end{array}\right.$$

Activated T cells have a mean lifespan 1/*b* and secrete IL-2 (*I*) with rate *d*. Available IL-2 decays with rate *f* and is consumed by activated T cells with rate *e*. Activated T cells are able to proliferate (with rate *aI*) in the presence of IL-2. This IL-2 dependent proliferation rate is considered as a linear function of IL-2 in model (1). The impact of considering a nonlinear proliferation rate (a Hill-function of IL-2) instead of the linear term *aIT* is given in Section “Nonlinear Proliferation Rate of Conventional and Regulatory T Cells” in Appendix.

Steady state analysis of the model (1) is given in Section “Steady State Analysis of Model (1)” in Appendix. This model has two equilibrium points:

(2) $$\left(T_{1},I_{1}\right)=\left(0,0\right),\left(T_{2},I_{2}\right)=\left(\frac{bf}{ad-be},\frac{b}{a}\right)$$

By assuming the biological range of parameters (all parameters are positive), the trivial equilibrium point (*T*_1_, *I*_1_) is stable and the nontrivial equilibrium (*T*_2_, *I*_2_) is unstable. *T*_2_ is positive if and only if:

(3) ad−be>0

**Figure 3:**
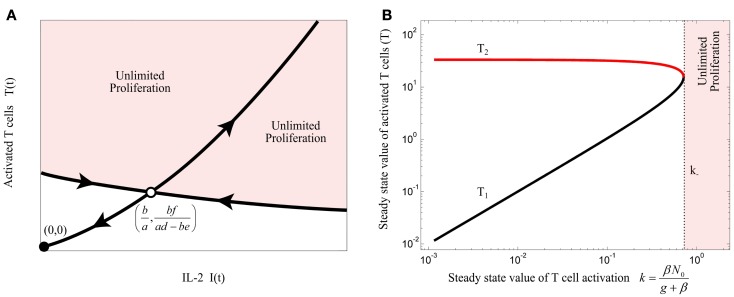
**(A)** Qualitative phase portrait of model (1): the stable manifold of saddle node defines a threshold for the initial conditions that allow for unlimited proliferation of activated T cells. **(B)** Bifurcation diagram of model (4) by treating *k* as bifurcation parameter. Stable and unstable equilibrium points are shown by black and red lines, respectively. For *k* > *k*_−_, the immune response enters the regime of unlimited proliferation.

The unstable equilibrium point imposes a threshold for initial conditions of the model in which the activated T cells proliferate unlimitedly, which in this simplest model, corresponds to an efficient immune response. This can be visualized by the phase portrait of the model as shown in Figure 3A. The condition (3) imposes a quality constraint on activated T cell clones to enter a highly proliferative state and implies that among T cell clones that are in the activated state, only the T cell clones with a sufficiently high proliferation rate (*a*) or IL-2 secretion rate (*d*) are able to contribute to the immune response against Ag. Since both, the proliferation and IL-2 secretion rate of activated T cells depend on the affinity/avidity of their TCR to the presented Ag (32–34), condition (3) implies that the existence of T cell clones with sufficiently high specificity for the presented Ag is required for induction of an immune response. Similar implications were derived from a model that considers a nonlinear IL-2 dependent proliferation rate of activated T cells (Nonlinear Proliferation Rate of Conventional and Regulatory T Cells in Appendix).

The major focus of central tolerance is to eliminate T cells that are self-specific. Therefore, it is unlikely that highly self-specific T cells escape from central tolerance, as they are more effectively detected and eliminated in the thymus (12, 34). It is thus expected that autoreactive T cells in the periphery are less aggressive than the ones that undergo clonal deletion in the thymus, and may not fulfill condition (3).

### Initiation of an immune response requires a minimum homeostatic population of naïve T cells and antigen stimulation

Continuous thymic production of naïve T cells maintains the peripheral number and diversity of mature naïve T cells (35), although other mechanisms such as stimulation of T cells with self-antigens and IL-7 have been shown to be involved (36). Upon Ag-stimulation by activated APCs, naïve T cells with high avidity to the presented Ag become activated. Here, we take into account the dynamics of the naïve T cell population (*N*) and T cell activation by Ag-stimulation (*β*), as described in equations (4). We assume that naïve T cells with identical Ag-specificity have a homeostatic population in the periphery that is established by naïve T cell renewal (by rate *N*_0_) and natural cell death (with rate *g*):

(4) $$\left\{\begin{array}{c} \frac{dN}{dt}=f\left(N\right)=N_{0}-gN-\beta N\;\\ \frac{dT}{dt}=aIT-bT+\beta N\;\\ \frac{dI}{dt}=dT-eIT-fI\;\\ \; \end{array}\right.$$

T cell activation *k*(*t*) is defined as

(5) k(t)=βN(t)

Steady state analysis of model (4) is given in Section “Steady State Analysis of Model (4)” in Appendix. This model has either 2 or no equilibrium points dependent on the steady state value of T cell activation (*k*). According to the bifurcation diagram of the model depicted in Figure 3B, which is obtained by treating *k* as bifurcation parameter, model (4) has no equilibrium points for:

(6) $$k>k_{-}=\frac{adf}{e^{2}}{\left(1-\sqrt{1-\frac{be}{ad}}\right)}^{2}$$

which corresponds to the unlimited proliferation state of activated T cells. Therefore, condition (6) has to be satisfied for initiation of an immune response. However, according to model (4), the steady state value of T cell activation (*k*) is limited by naïve T cell renewal (*N*_0_) and Ag-stimulation (*β*):

(7) $$k=\frac{\beta N_{0}}{g+\beta }$$

Therefore, according to equations (6) and (7), there exists an Ag-stimulation range

(8) $$\beta >\frac{gk_{-}}{N_{0}-k_{-}}$$

in which an immune response is initiated if:

(9) $$N_{0}>k_{-}$$

Condition (9) implies that the renewal rate of naïve T cells plays a critical role for the initiation of immune responses. In other words, without a sufficient renewal rate of naïve T cells, the immune response cannot be initiated by any Ag-stimulation. Instead, Ag-stimulation results in a subcritical immune response which is interpreted as insufficient for pathogen clearance. By increasing the proliferation rate or IL-2 secretion of activated T cells or the renewal rate of naïve T cells, the threshold of Ag-stimulation required for initiation of an immune response is decreased [equations (6) and (8)]. Therefore, central tolerance is able to tune the initiation criterion of self reaction not only by limiting the quality of autoreactive T cells, but additionally by restricting the renewal rate of autoreactive T cells. As central tolerance does not limit nonself-specific T cells, according to the model, these cells exhibit a lower threshold of activation by nonself Ag-stimulation.

### Fratricide: A mechanism to limit but not to suppress immune responses

The immune response in model (4) is characterized by unlimited proliferation of activated T cells which is physiologically unrealistic. The linear death term of natural death of activated T cells in model (4) is not sufficient to limit proliferation, and requires a nonlinear limiting factor. A potential mechanism of limiting the immune response is activation-induced cell death (AICD) in activated T cells, known as fratricide (37). Upon T cell activation, death ligand (FasL) and receptor (Fas) proteins are expressed on the surface of T cells. Followed by expression of these proteins, T cells eliminate themselves in a cell-contact-dependent manner. The fratricide mechanism is modeled by a nonlinear death term (*cT*^2^), as proposed by Callard et al. (37):

(10) $$\left\{\begin{array}{c} \frac{dN}{dt}=f\left(N\right)=N_{0}-gN-\beta N\;\\ \frac{dT}{dt}=aIT-bT-cT^{2}+\beta N\;\\ \frac{dI}{dt}=dT-eIT-fI\;\\ \; \end{array}\right.$$

**Figure 4:**
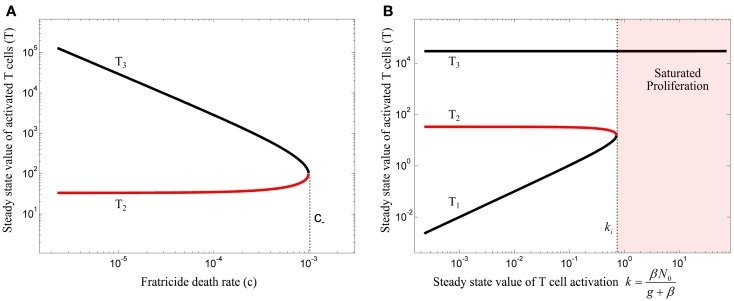
**(A)** Bifurcation diagram of model (10) with *β* = 0 using the fratricide death rate *c* as bifurcation parameter. No immune response exists for fratricide death rates larger than *c*_−_ due to extensive activation-induced cell death. The trivial equilibrium point is omitted in this figure. **(B)** Bifurcation diagram of model (10) using *k* as bifurcation parameter: an immune response can be initiated for large values of *k*. However, due to hysteresis characteristic in this model, the immune response is not suppressed after decreasing T cell activation (*k*). Stable and unstable equilibrium points are shown by black and red lines, respectively.

The steady state analysis of model (10) is provided in Section “Steady State Analysis of Model (10)” in Appendix. This model has either 3 or 1 equilibrium points, depending on the value of fratricide death rate *c*. The bifurcation diagram of the model (10) with respect to *c* is depicted in Figure 4A for (*β* = 0). When *c* satisfies

(11) $$c<c_{-}=f^{-1}{\left(\sqrt{ad}-\sqrt{be}\right)}^{2}$$

the stable equilibrium point (*T*_3_) exists and corresponds to a saturated population of activated T cell. When the conditions (3) and (11) are fulfilled, the model (10) exhibits the bifurcation diagram plotted in Figure 4B with respect to the steady state value of T cell activation (*k*). The fratricide mechanism added a large stable equilibrium point (*T*_3_) to the model which imposes a saturation level to the activated T cell population. The larger the *c*, the smaller the saturated population of activated T cells is. Similar to model (4), model (10) shows an initiation threshold of the immune response (*k* > *k_i_*). Despite solving the issue of unlimited proliferation of activated T cells by the fratricide mechanism, model (10) bears a hysteresis characteristic so that the immune response cannot be suppressed when Ag-stimulation (*β*) is removed.

### Dynamic interplay of activated T cells and Tregs

Tregs are essential in maintaining self-tolerance and immune homeostasis by preventing autoimmunity and limiting chronic inflammation in the periphery. However, they might also limit beneficial responses by inducing tolerance to pathogens (38, 39) or limiting anti-tumor immunity (40, 41). One functional role of Tregs is to shut down the cell-mediated immune response via cell-contact-dependent and inhibitory cytokine-driven suppression of activated T cells (42). Two different subsets of Tregs were identified. Natural Tregs are the dominant subset of peripheral Tregs (43) and are selected in the thymus. In our model, we consider only natural Tregs and neglect the induced Treg subset that differentiates from naïve T cells. Like for naïve T cells, the thymus contributes to the renewal of resting Tregs $\left(\hat{N}\right)$ by continuously selecting them from thymocytes. The renewal of resting Tregs is assumed to occur by rate ${\hat{N}}_{0}$. Since we are interested in the relative renewal of resting Tregs and naïve T cells, we assume that:

(12) $${\hat{N}}_{0}=\lambda N_{0}$$

Tregs remain in the resting state until they are stimulated by Ag (*β*) and become activated in a TCR-dependent manner. The dynamic population of the resting Treg compartment is assumed to be the same as the naïve T cell compartment in (4) and (10) $\left(d\hat{N}∕dt=f\left(\hat{N}\right)\right)$. Activated Tregs (*R*) are assumed to suppress activated T cells (by rate γ). Survival and proliferation of activated Tregs depends strictly on IL-2, produced by activated non-Tregs (44–46). The IL-2 dependent proliferation rate of Tregs is considered as a linear function of IL-2 (see Nonlinear Proliferation Rate of Conventional and Regulatory T Cells in Appendix for a nonlinear case). In contrast to activated T cells, activated Tregs lack the ability to secrete IL-2 (47). The relative proliferation rate of activated Tregs and activated T cells is controlled by the parameter ϵ:

(13) $$\left\{\begin{array}{c} \frac{dT}{dt}=aIT-bT-cT^{2}-\gamma RT+\beta N\;\\ \frac{dR}{dt}=\epsilon aIR-bR+\beta \hat{N}\;\\ \frac{dI}{dt}=dT-eI\left(T+R\right)-fI\;\\ \; \end{array}\right.$$

The parameters are given in Table 1 and the model components are illustrated in Figure 2. Treg activation $\hat{k}\left(t\right)$ is defined as

(14) $$\hat{k}\left(t\right)=\beta \hat{N}\left(t\right)$$

**Table 1:** **Parameters used for model analysis**.

Parameter	Value	Description	Dimension
*a*	0.4	Proliferation rate of activated T cells	molecules^−1^time^−1^
*b*	0.1	Natural death rate of activated T cells and Tregs	mime^−1^
*c*	10^−5^	Fratricide death rate of activated T cells	cells^−1^time^−1^
*d*	0.01	IL-2 secretion rate by activated T cells	molecules cells^−1^time^−1^
*e*	0.01	IL-2 consumption rate by activated T cells and Tregs	cells^−1^time^−1^
*f*	1	IL-2 decay rate	time^−1^
*g*	B	Natural death rate of naïve T cells and resting Tregs	time^−1^
*β*	[0,∞)	Ag-stimulation of naïve T cells and resting Tregs	time^−1^
γ	0.1	Treg-mediated suppression rate	cells^−1^time^−1^
ϵ	0.6	Proliferation rate ratio Treg/Tconv	–
*N*_0_	4	Renewal rate of naïve T cells	cells time^−1^
λ	0.006, 0.02	Relative renewal rate of resting Tregs and naïve T cells ${\hat{N}}_{0}∕N_{0}$	–
${\hat{N}}_{0}$	λ *N*_0_	Renewal rate of resting Tregs	cells time^−1^

According to equations (7) and (12), the steady state value of Treg activation $\left(\hat{k}\right)$ is given by

(15) $$\hat{k}=\lambda \frac{\beta N_{0}}{g+\beta }=\lambda k$$

**Figure 5:**
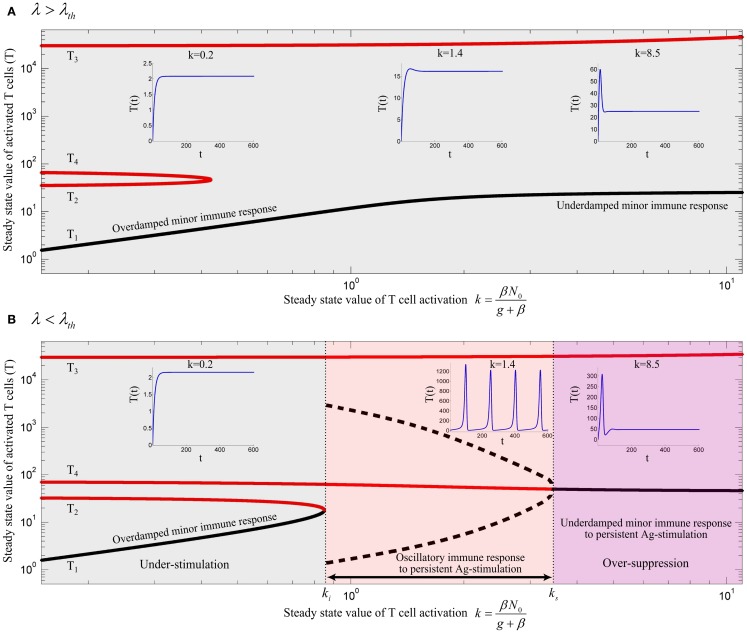
**Bifurcation diagram of model (13) using *k* as the bifurcation parameter with (A) λ = 0.02 and (B) λ = 0.006**. Stable and unstable equilibrium points are drawn by black and red solid lines, respectively. Dashed black lines represent the stable limit cycles by showing the maximum and minimum populations of oscillating activated T cells for persistent *k*. Depending on the values of λ and *k*, an immune response is not initiated (gray), is initiated (red) or over-suppressed (magenta). With parameter values given in Table 1, the threshold becomes λ_th_ = 0.01183. The time-courses of the activated T cell population *T*(*t*) were deduced from a numerical solution of model (13) with zero initial conditions and persistent *β*. The unstable negative equilibrium point (*T*_5_) is not shown in the plots.

The equilibrium points of model (13) are given in Section “Steady State Analysis of Model (13)” in Appendix. By incorporating the Treg compartment to model (10), two additional equilibrium points (*T*_4_ and *T*_5_) emerged for *β* = 0. The equilibrium point of interest (*T*_4_), which depends on the Treg-associated parameters (*ϵ, γ*), has an impact on the topological changes of the phase portraits of the model under variations of the bifurcation parameter *k*. The value of *ϵ* and *γ* are assumed to be in a range where the model does not inherit the hysteresis characteristics of immune responses from model (10) in which the immune response is not suppressed after resolving Ag-stimulation (*β*). Then, the bifurcation diagrams of model (13) for two different values of *λ* are obtained by treating *k* as the bifurcation parameter (Figure 5). Depending on the value of *k*, the model has either 5 or 3 equilibrium points.

By varying the relative renewal rates of resting Tregs and naïve T cells [*λ* in equation (12)] a *λ*_th_ can be found, so that no immune response can be initiated for any value of *k*, if *λ* > *λ*_th_ (Figure 5A). For *λ* < *λ*_th_ (Figure 5B), there exists a T cell activation threshold (*k_i_*) such that for *k* > *k_i_* the immune response can be initiated. However, in contrast to model (10), the immune response is completely suppressed by activated Tregs if *k* decreases to a lower value than *k_i_* (gray region in Figure 5B). For persistent Ag-stimulation with *k* > *k_i_*, two scenarios are possible. An oscillating immune response is induced when *k* remains in the range of *k_i_* < *k* < *k_s_* (red region in Figure 5B). For *k* > *k_s_* the immune response is suppressed after its initiation to a minor immune response with an activated T cell population *T*_4_ due to over-suppression of activated T cells by over-activation of Tregs (magenta region in Figure 5B). In the latter case (*k* > *k_s_*), despite proper T cell stimulation, only a minor immune response is induced (and antigen is not cleared). Instead a chronic co-existence of antigen and inefficient immune activity is established. Therefore, according to the model, a range of T cell and Treg activation (*k_i_* < *k* < *k_s_*) exists in which an efficient immune response is induced. Outside of this range, the antigen persists because of under-stimulation of naïve T cells, or over-stimulation of Tregs. According to equation (7), the existence of Ag-stimulation thresholds *β_i_* and *β_s_* which correspond to the values of *k_i_* and *k_s_*, respectively, depends on the renewal rate of naïve T cells (*N*_0_); *β_i_* exists if *N*_0_ > *k_i_* and *β_s_* exists if *N*_0_ > *k_s_*. Increasing the renewal rate of naïve T cells reduces the Ag-stimulation required for initiation(*β_i_*)/over-suppression(*β_s_*) of the immune response.

The peak immune response depends on the value of the Treg-associated equilibrium point (*T*_4_) which in turn depends on Treg-associated parameters. However, the fratricide-associated equilibrium point (*T*_3_) is a limiting factor for the maximum population of activated T cells if the fratricide death rate (*c*) is sufficiently high.

According to our model, sufficient activated Tregs are required to suppress the proliferative response of activated T cells. These are supplied by two processes: Treg activation $\left(\hat{k}\right)$ which depends on Ag-stimulation (*β*), and Treg proliferation which depends on the IL-2 secretion by activated T cells. With a low Ag-stimulation and insufficient Treg activation $\left(\hat{k}=\beta \hat{N}\right)$, Treg proliferation has to account for immune suppression. Since Treg proliferation is dependent on the availability of IL-2, sufficient activated T cells are required to secrete IL-2 and induce immune suppression. Therefore, activated T cells undergo the proliferation up to a level that sufficient IL-2 is available for Treg proliferation and subsequent immune suppression. In contrast, by facilitated Treg activation $\left(\hat{k}\right)$, less Treg proliferation is required for suppressing activated T cells which means that the dependency of immune suppression on proliferation of activated T cells decreases. Consequently, by increasing Ag-stimulation (*β*) in the range of *β_i_* < *β* < *β_s_* (red region in Figure 5B), Treg activation $\left(\hat{k}\right)$ increases as well which results in a reduced maximum population of activated T cells (Figure 5B, dashed black line) and an increased frequency of oscillations. By further increasing Ag-stimulation to *β* > *β_s_* (magenta region in Figure 5B), Treg activation $\left(\hat{k}\right)$ completely prevent oscillating immune response.

In the same way, by increasing the relative homeostatic population of resting Tregs and naïve T cells (*λ* > *λ*_th_), Treg activation increases up to a level that Treg suppression does not depend on the proliferative response of activated T cells. Thus, activated T cells are not able to enter the massive proliferation for any Ag-stimulation level, as shown in Figure 5A. Similar results were derived from a model that considers a nonlinear IL-2 dependent proliferation rate of activated T cells and Tregs (see Nonlinear Proliferation Rate of Conventional and Regulatory T Cells in Appendix).

## Discussion

In this study, a model of the dynamic interplay between effector and regulatory immune responses was examined to investigate the requirements for the initiation of an immune response by Ag-stimulation. The model unifies several components developed in previous studies, such as IL-2 dependent proliferation of T cells (48), fratricide-induce programed cell death (37), IL-2 competition between activated T cell and activated Tregs (24), and Treg-mediated immune suppression (23, 24, 28). Homeostatic division of T cell compartments was not considered in the present study, such that the main renewal source of T cells in the absence of Ag-stimulation is the thymus. While the presented model is still simplifying the real situation in many aspects, the stability analysis revealed a number of reasonable results matching many experimental findings and allowing for an analysis of reasons for immune failure and autoimmunity.

The model predicts three qualitatively different immune responses depending on the level of antigenic stimulation. At first, a threshold stimulation *β_i_* is required in order to get an immune response at all. Secondly, in a limited range of Ag-stimulation *β* ∈ (*β_i_, β_s_*) an efficient immune response is induced. Tregs limit the duration of the immune response. If the antigen was cleared by the first immune response, further immune activity would be suppressed by Tregs. However, if the first peak of the immune response fails to clear the antigen, but keeps the antigen in the stimulation range *β_i_* < *β* < *β_s_*, the immune system attempts to clear the antigen with subsequent immune responses, which corresponds to the oscillatory solution depicted in Figure 5B. If the immune response failed to control the antigen spread, antigenic stimulation would be further increased to *β* > *β_s_*, leading to the third class of immune responses. Tregs are over-stimulated and suppress immune activity. In this situation, a chronic persistence of the antigen would develop. Treg-mediated over-suppression of immune responses in chronic infections is well-established (reviewed in Ref. (49)). According to our model, depletion of resting Tregs restores the immune response by transiently decreasing *λ* and by this increasing *β_s_*. This notion is consistent with the experimental model of chronic infections according to which depletion of Tregs results in the restoration of effector immune response and restriction of antigen spread (50, 51). A key feature of our model is that the immune response does not rely on a stable equilibrium point with a dominant population of activated T cells which is typically derived from existing bistable models. It rather relies on a transient response (or stable limit cycles in the case of persistent Ag-stimulation) which originates from T-cell-mediated suppression and IL-2 consumption by Tregs. Moreover, the role of Tregs in the chronic state of the immune response is not represented by available models.

According to our model, the qualitatively different immune responses and their requirements are dependent on the quality and quantity of Tconv and Treg clones responding to the Ag-stimulation. The proliferation rate of activated T cells, which depends on their avidity to the stimulating antigen determines the existence of an Ag-stimulation threshold (*β_i_*) which is required for the initiation of an immune response. The absolute renewal rate of naïve T cells (*N*_0_) adjusts the Ag-stimulation threshold *β_i_*, which exists when the renewal rate of resting Tregs remains below a threshold value (*λ* < *λ*_th_). Further Treg-associated parameters, namely the proliferation rate of Tregs (*ϵ*) and the Treg-mediated suppression rate (*γ*), also affect the existence and the level of the Ag-stimulation required for initiation (*β_i_*) and over-suppression (*β_s_*) of immune responses. By increasing the proliferative (*ϵ*) and suppressive (*γ*) activity of Tregs, *β_i_* increases, whereas *β_s_* decreases up to a level where the initiation of an immune response is completely impossible for any Ag-stimulation. Interestingly, when proliferation rate of activated Tregs exceeds the one for activated T cells (*ϵ* > 1) a massive proliferation of activated T cells is still required for subsequent immune suppression by Tregs. Thus, IL-2 secretion by sufficiently large numbers of activated T cells is a strict requirement for immune suppression. Note also that without Tregs, a return to the homeostatic state is not possible, even when the antigen was cleared.

**Figure 6:**
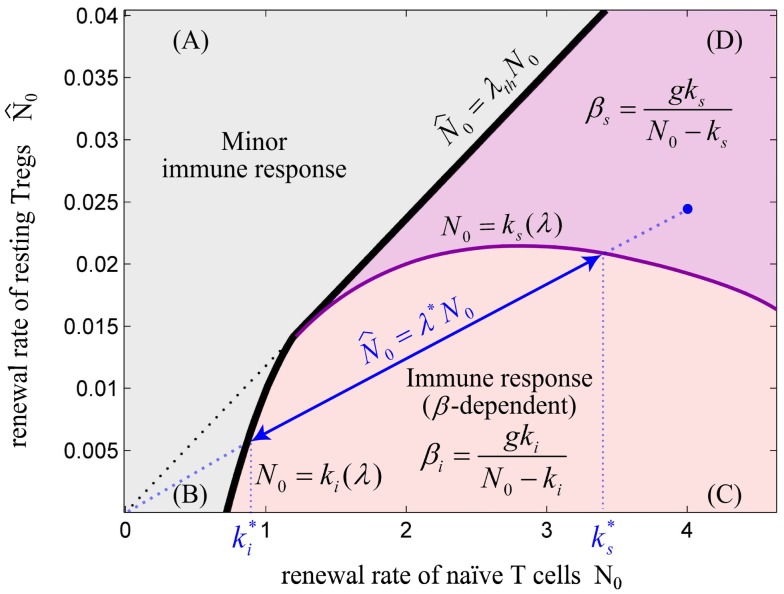
**The balance between renewal rate of naïve T cells and resting Tregs**. The relative renewal rate of naïve T cells and resting Tregs $\left(\lambda ={\hat{N}}_{0}∕N_{0}\right)$ determines the existence of an immune response. The initiation of an immune response requires a sufficient renewal rate of naïve T cells (*N*_0_). **(A)** For λ > λ_th_, the immune response does not exist for any value of *N*_0_ and Ag-stimulation (*β*). **(B)** For *N*_0_ < *k_i_*, no immune response can be initiated for any value of *β*. **(C)** For *N*_0_ > *k_i_*, immune response can be initiated for *β* > *β_i_*. In this regime, the Ag-stimulation that results in over-suppression of immune response (*β_s_*) does not exist. **(D)** In this regime, immune response is initiated for *β* > *β_i_*, and is over-suppressed for *β* > *β_s_*. Note that *k_i_* and *k_s_* are dependent on the value of λ. For any point in the plane (e.g., blue point), the values of $k_{s}^{*}$ and $k_{i}^{*}$ are obtained by projecting the intersections of the line created by connecting the point to the origin (slope = λ*) with the nonlinear curves *N*_0_ = *k_i_*(λ) and *N*_0_ = *k_s_*(λ) onto the *N*_0_-axis. By decreasing λ*, the effective range of T cell activation $\left(k_{s}^{*}-k_{i}^{*}\right)$ or equivalently, the effective range of Ag-stimulation $\left(\beta_{s}^{*}-\beta_{i}^{*}\right)$ which evoke immune response without over-suppression increases. We hypothesize that a healthy individual bears the potential to evoke self reaction and therefore its immune system is located in parametric regime **(C)** or **(D)**; however, higher self Ag-stimulation compared to nonself Ag-stimulation is required for initiating immune response due to low renewal rate of autoreactive T cells (*N*_0_).

Considering all aforementioned parameters controlling the initiation of an immune response, is it beneficial for the immune system to completely avoid self reaction, or is there a benefit in allowing self reaction? Clearly, autoreactive T cells exist in the periphery of healthy individuals as a normal component of the T cell repertoire (12, 14, 52, 53). These cells respond to self-tissue destruction even in the presence of Tregs and without genetic predisposition to autoimmunity (15). Although the activation of autoreactive T cells has been shown to be involved in autoimmunity (12), several lines of evidences indicate that these cells are required for limiting self-destruction by supporting self-regenerative processes (54–56). In addition, the anti-tumor immune responses evoked by autoreactive T cells are beneficial (34, 57). Therefore, it seems unlikely that autoreactive T cells escaping from the thymus are simply a result of thymic selection error that can disturb self-tolerance under certain physiological conditions. Instead, these evidences imply that beneficial self reaction is allowed in the immune system. According to the mathematical model, immune reactions against self are only possible with a critical homeostatic population of autoreactive T cells (or sufficient renewal rate *N*_0_) which is balanced by a proper number of Tregs (*λ* < *λ*_th_) which corresponds to region (C) or (D) in Figure 6. Since the T cell repertoire is normally stimulated with an endogenous level of self-antigens in the periphery which does not evoke any self reaction, the Ag-stimulation threshold for initiating an immune response (*β_i_*) should be sufficiently high in comparison to a typical nonself Ag-stimulation. According to our model, this is achieved by ensuring a low renewal rate (*N*_0_) of low-avidity autoreactive T cells and a high, but balanced renewal rate of Tregs (high *λ* but lower than *λ*_th_). In other words, according to Figure 6, by choosing *N*_0_ close to *k_i_* and higher value of *k_i_* which is obtained by higher *λ*, a large Ag-stimulation threshold (*β_i_*) for the initiation of immunity against self can be achieved.

Aging of the immune system, the so-called immunosenescence, is characterized by involution of thymus, decreased number of thymic output, contraction in T cell diversity, and disturbed T cell homeostasis which all result in attenuated immune function and susceptibility to infectious diseases and cancer in the elderly (58, 59). By decreasing thymic output, the homeostatic population of some T cell clones diminishes which leads to the creation of holes in the T cell repertoire (60). According to our model, a decreased renewal rate of a naïve T cell clone (*N*_0_) *per se* could prevent an immune response or increase the Ag-stimulation level required for initiation of an immune response. In addition, as shown in many studies, the frequency of Tregs increases with age (61, 62) which results in a disturbed balance between the population of naïve T cells and resting Tregs (increased *λ*). In line with these results, in the mathematical model an increased *λ* prevents the initiation of an immune response corresponding to the age-related immune hyporesponsiveness in infection and cancer.

Based on the reasonable and physiologically realistic results that we could derive from the model, we dare to speculate about the self versus nonself concept emerging from the model. As mentioned before, the naïve T cells and resting Tregs are two major components of the immune reaction. The model does not distinguish self and nonself, but rather derives different responses to self and nonself from quantitative differences in the nature of Ag-stimulation. According to the model, by adjusting different parameters, different requirements in terms of Ag-stimulation level are found for the initiation of immune responses to self versus nonself. If the immune system responds according to a universal set of Ag-stimulation thresholds, regardless of whether the stimulus arises from self or nonself-antigens, a change of systemic parameters can lead to immune failure or autoimmunity. Self is no more considered as self if it exceeds an Ag-stimulation threshold determined by the stringency of central and peripheral tolerances. Similarly, nonself is considered as self if it does not properly stimulate the T cell repertoire. Autoimmunity might occur due to either a failure in tuning the Ag-stimulation threshold by the thymus that leads to unwanted self reaction in the periphery, or a chronic self Ag-stimulation in the periphery that leads to an oscillating self reaction and tissue destruction like in type 1 diabetes (63) and multiple sclerosis (64). Cancer or chronic infection would arise as the result of an imbalance in central and peripheral tolerances such as insufficient release of autoreactive T cells as well as high production or induction of Tregs that results in over-suppression of immune responses.

An early elegant mathematical modeling study analyzed a series of models to investigate self/nonself discrimination by T cells without explicitly considering suppressive Tregs (48). As a result of their critical assumption that memory cells accumulate in poor stimulatory conditions, the authors suggested that due to high stimulation by self antigens the lack of memory accumulation for T cell clones with high affinity to self accounts for self-tolerance. Also in our model, a high self Ag-stimulation (*β* > *β_s_* in Figure 5B) results in over-activation of Tregs and by this in over-suppression of self reaction. In both models an increased stimulation by self antigen would not lead to autoimmunity. The fact that autoreactive T cells do respond in the presence of Tregs when their stimulatory requirements are provided (15) makes it unlikely that this is the mechanism of self-tolerance induction. In the framework of our model, the view is supported that immune tolerance is induced by an increased stimulation threshold for self antigen and keeping self Ag-stimulation in a subcritical regime (*β* < *β_i_*).

Undoubtedly, other mechanisms besides clonal deletion and Treg selection in the thymus also contribute to the fine tuning of the Ag-stimulation threshold required for initiation of immune reactions to self and nonself, such as anergy in the periphery (65) or activation threshold tuning in the thymus (66, 67). However, our simple model emphasizes the subtle balance between the generation of Tregs and autoreactive T cells which are both needed for beneficial autoimmunity. The model supports the view according to which self and nonself do not differ on a qualitative level. It is rather quantitative differences of the immune status and Ag-stimulation level that determine which molecule is treated as self or nonself.

## Conflict of Interest Statement

The authors declare that the research was conducted in the absence of any commercial or financial relationships that could be construed as a potential conflict of interest.

## Figures and Tables

**Figure A1:**
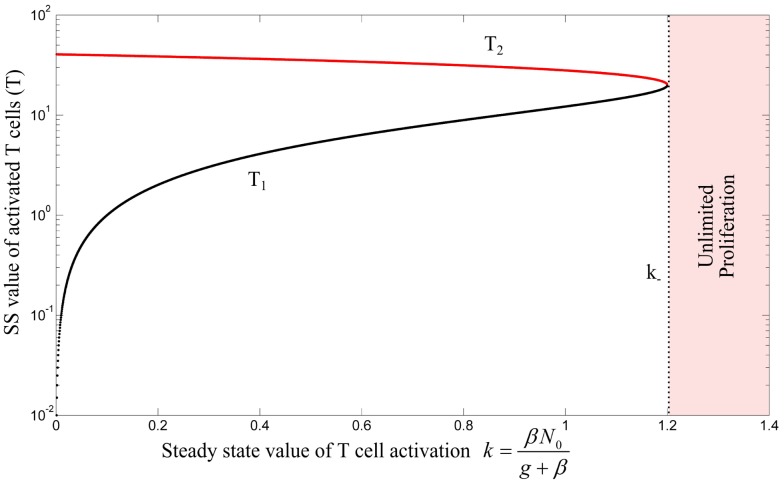
**Bifurcation diagram of model (A63) with Hill-function parameters *n* = 2 and h = 0.5 by treating k as bifurcation parameter**. Stable and unstable equilibrium points are shown by black and red lines, respectively. For *k* > *k*_−_, the immune response enters the regime of unlimited proliferation. The unstable negative equilibrium point is omitted in this figure.

**Figure A2:**
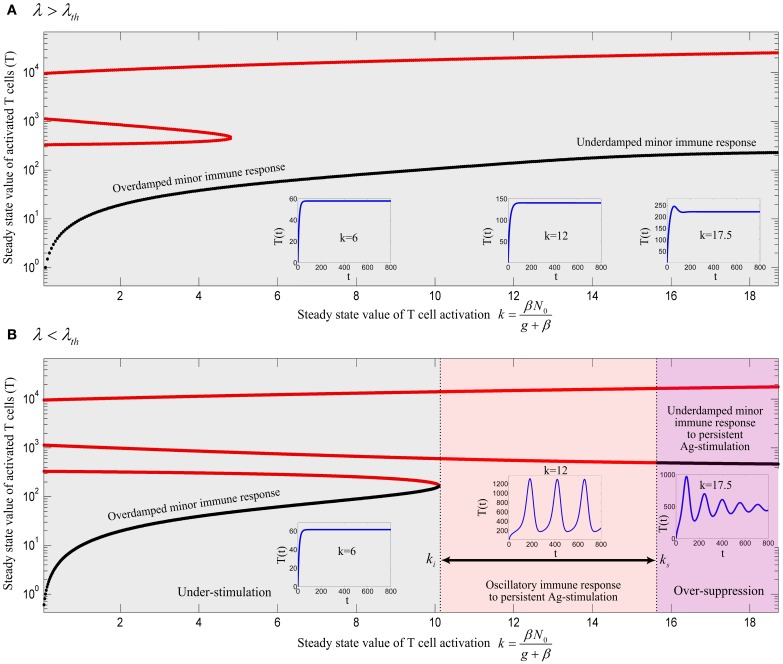
**Bifurcation diagram of model (A73) with Hill-function parameters *n* = 4 and h = 1 using *k* as the bifurcation parameter with (A) λ = 0.0016 and (B) λ = 0.0008**. Stable and unstable equilibrium points are drawn by black and red solid lines, respectively. The stable limit cycles are not shown for all value of *k_i_* < *k* < *k_s_* except for *k* = 12. Depending on the values of λ and *k*, an immune response is not initiated (gray), is initiated (red) or over-suppressed (magenta). With parameter values given in Table 1 and Hill-function parameters *n* = 4 and h = 1, the threshold becomes λ_th_ = 0.00111. The time-courses of the activated T cell population *T*(*t*) were deduced from a numerical solution of model (A73) with zero initial conditions and persistent *β*. The negative equilibrium points which are all unstable are not shown in the plots.

## Appendix

### A.1 Steady state analysis of model (1)

The equilibrium points of model (1) can be obtained from the following:

(A1) $$\mathrm{Equilibrium}\;\mathrm{points}=\left\{\begin{array}{c} \left(T_{1},I_{1}\right)=\left(0,0\right)\;\\ \left(T_{2},I_{2}\right)=\left(\frac{bf}{ad-be},\frac{b}{a}\right)\;\\ \; \end{array}\right.$$

For a positive nontrivial equilibrium point (*T*_2_, *I*_2_), we have to assume that:

(A2) ad−be>0

The stability of equilibrium points can be determined from the sign of real part of eigenvalues of Jacobian matrix (*J*). An equilibrium point is stable if all the eigenvalues of *J* evaluated at the equilibrium point have negative real parts, and it is unstable if at least one of the eigenvalues has a positive real part.

(A3) $$\mathrm{Jacobian}\;\mathrm{Matrix}\;J=\left[\begin{array}{cc} aI-b & aT\\ d-eI & -eT-f \end{array}\right]$$

(A4) $$\begin{array}{ccc} \mathrm{Characteristic}\;\mathrm{Equation}\;Q\left(\lambda \right) & =det\left\{\left[\begin{array}{cc} \lambda -aI+b & -aT\\ -d+eI & \lambda +eT+f \end{array}\right]\right\} & \\ & =\lambda^{2}+\left[eT+f+b-aI\right]\lambda +\left[-af\;I+beT+bf-adT\right]=0 \end{array}$$

The eigenvalues (*λ*_1,2_) of *J* for trivial equilibrium point (*T*_1_, *I*_1_) are obtained by solving the characteristic equation (A4):

(A5) $$\lambda_{1,2}{|}_{\;(T_{1,}I_{1})}:Q(\lambda ){|}_{\left(T_{1,}I_{1}\right)}=\lambda^{2}+\left(f+b\right)\lambda +bf=0$$

By checking Routh–Hurwitz stability Criterion (RHC) it can be easily confirmed that the eigenvalues have negative real parts since all the coefficients of polynomial *Q*(λ) are positive, and hence, the trivial equilibrium point (*T*_1_, *I*_1_) is locally stable. For stability of nontrivial equilibrium point, the characteristic equation (A4) is evaluated and solved in (*T*_2_, *I*_2_):

(A6) $$\lambda_{1,2}{|}_{\left(T_{2,}I_{2}\right)}:Q(\lambda ){|}_{\left(T_{2,}I_{2}\right)}=\lambda^{2}+\left(\frac{afd}{ad-be}\right)\lambda -bf=0$$

With the assumption (A2), the coefficient of *λ* is positive. The sign of *Q*(*λ*) coefficients change only once and hence, there exists one positive eigenvalue. Therefore, the nontrivial equilibrium point (*T*_2_, *I*_2_) is a saddle point and unstable.

### A.2 Steady state analysis of model (4)

The equilibrium points of model (4) with definition of T cell activation (*k*(*t*)) given in equation (5) can be obtained from the following

(A7) $$\begin{array}{ll} & N=\frac{N_{0}}{g+\beta }=\frac{k}{\beta } \end{array}$$

(A8) $$\begin{array}{ll} & I=\frac{dT}{eT+f}=\frac{bT-k}{aT} \end{array}$$

(A9) $$\begin{array}{ll} & \left(ad-be\right)T^{2}+\left(ek-bf\right)T+fk=0 \end{array}$$

By keeping the assumption (A2), if the coefficient of *T* in (A9) is negative, the equilibrium points (*T*), if exist, will be positive:

(A10) $$ek-bf<0\to k<\frac{bf}{e}$$

Otherwise, the equilibrium points will be negative. According to equation (A8), for *T* ≥ 0, *I* ≥ 0. Now, let’s check the condition for existence of equilibrium points:

(A11) $$\begin{array}{ll} \Delta =0\to {\left(ek+bf\right)}^{2}-4afdk & =\underset{>0}{\underset{︸}{\left(ek+bf+2\sqrt{afdk}\right)}}\underset{=0}{\underset{︸}{\left(ek+bf-2\sqrt{afdk}\right)}}=0 \end{array}$$

(A12) $$\begin{array}{ll} \left(ek+bf-2\sqrt{afdk}\right)=0\to k_{+} & =\frac{adf}{e^{2}}{\left(1+\sqrt{1-\frac{be}{ad}}\right)}^{2},k_{-}=\frac{adf}{e^{2}}{\left(1-\sqrt{1-\frac{be}{ad}}\right)}^{2} \end{array}$$

The model does not have any equilibrium points for *k*_−_ < *k* < *k*_+_, and two equilibrium points otherwise. It can be verified that by keeping assumption (A2), we always have:

(A13) $$0<k_{-}<\frac{bf}{e}<k_{+}$$

Therefore, for 0 < *k* < *k*_−_, condition (A10) is satisfied and the model has two positive equilibrium points and for *k* > *k*_+_, condition (A10) is not satisfied and model has two negative equilibrium points. Let’s assume that the model has two positive equilibrium points (Δ > 0 and 0 < *k* < *k*_−_). In the following, the linear stability of equilibrium points is analyzed:

(A14) $$\mathrm{Jacobian}\;\mathrm{Matrix}\;J\;=\left[\begin{array}{ccc} -g-\beta & 0 & 0\\ \beta & aI-b & aT\\ 0 & d-eI & -eT-f \end{array}\right]$$

(A15) $$\begin{array}{ccc} \mathrm{Characteristic}\;\mathrm{Equation}\;Q\left(\lambda \right) & =det\left\{\left[\begin{array}{ccc} \lambda +g+\beta & 0 & 0\\ -\beta & \lambda -aI+b & -aT\\ 0 & -d+eI & \lambda +eT+f \end{array}\right]\right\} & \\ & =\left[\lambda +\left(g+\beta \right)\right]\underset{Q^{*}}{\underset{︸}{\left[\left(\lambda -aI+b\right)\left(\lambda +eT+f\right)+aT\left(eI-d\right)\right]}}=0 \end{array}$$

The model has one negative eigenvalue λ = −(*g* + *β*) for all equilibrium points. For the other two remaining eigenvalues, polynomial *Q** has to be checked for existence of positive eigenvalue.

(A16) $$Q^{*}=\lambda^{2}+\underset{U}{\underset{︸}{\left[eT+f+b-aI\right]}}\lambda +\underset{V}{\underset{︸}{\left[-af\;I+beT+bf-adT\right]}}=0$$

From equation (A8) it can be easily verified that *b* − *aI* > 0 and hence, coefficient *U* is positive. Therefore, the stability depends on the sign of coefficient *V*.

(A17) $$\begin{array}{ll} V & =-af\;I+beT+bf-adT\overset{I=\frac{dT}{eT+f}}{\to }V=-\frac{afd}{eT+f}T-\left(ad-be\right)T+bf \end{array}$$

(A18) $$\begin{array}{ll} V_{1} & =TV=-\frac{afd}{eT+f}T^{2}-\underset{\mathrm{According}\;\mathrm{to}\;\left(A9\right)\to =0}{\underset{︸}{\left[\left(ad-be\right)T^{2}+\left(ek-bf\right)T+fk\right]}}+k\left(eT+f\right) \end{array}$$

(A19) $$\begin{array}{ll} V_{2} & =\frac{1}{k\left(eT+f\right)}V_{1}=-\frac{af}{kd}{\left(\frac{dT}{eT+f}\right)}^{2}+11\overset{I=\frac{dT}{eT+f}}{\to }V_{2}=-\frac{af}{kd}I^{2}+1 \end{array}$$

According to equations (A8) and (A9), the equilibrium values of *I* are:

(A20) $$\begin{array}{ll} I_{2} & =\frac{1}{2}\left(\frac{\left(ek+bf\right)+\sqrt{\Delta }}{af}\right),T_{2}=\frac{k}{b-aI_{2}},N_{2}=\frac{N_{0}}{g+\beta } \end{array}$$

(A21) $$\begin{array}{ll} I_{1} & =\frac{1}{2}\left(\frac{\left(ek+bf\right)-\sqrt{\Delta }}{af}\right),T_{1}=\frac{k}{b-aI_{1}},N_{1}=\frac{N_{0}}{g+\beta } \end{array}$$

where *I*_2_ > *I*_1_ and:

(A22) $$\Delta ={\left(ek+bf\right)}^{2}-4f\;kad={\left(ek-bf\right)}^{2}-4f\;k\left(ad-be\right)$$

Next, the sign of *V*_2_, which is similar to *V*_1_ and *V*, has to be checked in the equilibrium points. For the larger equilibrium point (*T*_2_, *I*_2_):

(A23) $$\begin{array}{ccc} V_{2}{|}_{I_{2}} & =-\frac{1}{2f\;kad}\left[e^{2}k^{2}+2ekbf+ek\sqrt{\Delta }+b^{2}f^{2}+bf\sqrt{\Delta }-4f\;kad\right] & \\ & =-\frac{1}{2f\;kad}\underset{+}{\underset{︸}{\left[\Delta +\left(ek+bf\right)\sqrt{\Delta }\right]}}<0 \end{array}$$

*V*_2_, *V*_1_, and *V* are negative for (*T*_2_, *I*_2_). Therefore, the model in this equilibrium point has a positive eigenvalue and it is locally unstable. For (*T*_1_, *I*_1_),

(A24) $$V_{2}{|}_{I_{1}}=-\frac{\sqrt{\Delta }}{2f\;kad}{\left[\sqrt{\Delta }-\left(ek+bf\right)\right]}^{\underline{\underline{\text{According to (A22)}}}}\;-\frac{\sqrt{\Delta }}{2f\;kad}\underset{-}{\underset{︸}{\left[\sqrt{{\left(ek+bf\right)}^{2}-4f\;kad}-\left(ek+bf\right)\right]}}>0$$

*V*_2_, *V*_1_, and *V* are positive for (*T*_1_, *I*_1_). Therefore, all the eigenvalues of the model in this equilibrium point are negative and it is locally stable.

Next, let’s assume that *k* > *k*_+_ which means that the equilibrium points exist and the steady state values of *T* are negative, whereas the equilibrium values of *I* is positive. According to equation (A8), coefficient *U* in equation (A16) is negative since:

(A25) $$T=\frac{k}{b-aI}<0\to b-aI<0,I=\frac{dT}{eT+f}>0\overset{dT<0}{\to }eT+f<0$$

Therefore, the model at least has one positive eigenvalue in the equilibrium points. Let’s check the sign of coefficient *V* in the equilibrium points:

(A26) $$\begin{array}{ll} V & =-af\;I+beT+bf-adT^{\underline{\underline{T=\frac{k}{b-aI}}}}\;-f\left(aI-b\right)+\left(ad-be\right)\frac{k}{aI-b} \end{array}$$

(A27) $$\begin{array}{llll} V_{2} & =\left(aI-b\right)V^{\underline{\underline{aI-b>0}}}\;-f{\left(aI-b\right)}^{2}+\left(ad-be\right)k^{\underline{\underline{aI-b=-\frac{k}{T}}}}\;-f\frac{k^{2}}{T^{2}}+\left(ad-be\right)k\; & & \;\\ V_{3} & =\frac{T^{2}}{fk^{2}}V_{2}=-1+\left(\frac{ad-be}{fk}\right)T^{2} \end{array}$$

The sign of *V*_3_ in equation (A27) which is the same as the sign of *V* in equation (A16) can be determined by substituting *T* with its equilibrium values from equation (A9):

(A28) $$V_{3}{|}_{T_{2}}=\frac{1}{4\left(ad-be\right)fk}\underset{\mathrm{sign}?}{\underset{︸}{\left[2\Delta +2\sqrt{\Delta }\left(bf-ek\right)\right]}}$$

According to equation (A13) and reminding that *k* > *k*_+_, it can be verified that *V*_3_ or equivalently *V* is positive. Therefore, the equilibrium point (*T*_2_, *I*_2_) has one positive eigenvalue and is unstable. For (*T*_1_, *I*_1_),

(A29) $$V_{3}{|}_{T_{1}}=\frac{1}{4\left(ad-be\right)fk}\underset{+}{\underset{︸}{\left[2\Delta +2\sqrt{\Delta }\underset{+}{\underset{︸}{\left(ek-bf\right)}}\right]}}$$

*V*_3_ and equivalently, *V* are positive and therefore, the equilibrium point (*T*_1_, *I*_1_) has one positive eigenvalue and it is unstable.

### A.3 Steady state analysis of model (10)

The equilibrium points of model (10) for *β* = 0 can be obtained from the following

(A30) $$\begin{array}{ll} & \left(T_{1},\;I_{1},\;N_{1}\right)=\left(0,\;0,\;\frac{N_{0}}{g}\right) \end{array}$$

(A31) $$\begin{array}{ll} & N_{2,3}:N_{2,3}=\frac{N_{0}}{g} \end{array}$$

(A32) $$\begin{array}{ll} & T_{2,3}:ceT^{2}+\left(cf+be-ad\right)T+bf=0,\left\{\begin{array}{c} T_{3}=-\frac{1}{2ce}\left[-\left(cf+be-ad\right)+\sqrt{\Delta }\right]\;\\ T_{2}=-\frac{1}{2ce}\left[-\left(cf+be-ad\right)-\sqrt{\Delta }\right]\;\\ \Delta ={\left(cf+be-ad\right)}^{2}-4cf\;be\;\\ \; \end{array}\right. \end{array}$$

(A33) $$\begin{array}{ll} & I_{2,3}:I_{2,3}=\frac{cT_{2,3}+b}{a} \end{array}$$

According to equation (A32), the nontrivial equilibrium points, if available, are positive only if:

(A34) cf+be−ad<0

The condition of the existence of positive equilibrium points can be obtained from equation (A32):

(A35) $$\Delta ={\left(cf+be-ad\right)}^{2}-4cebf=f^{2}c^{2}-2f\left(ad+be\right)c+{\left(ad-be\right)}^{2}\geq 0$$

According to equation (A35), the nontrivial equilibrium points exist only if:

(A36) $$\left\{c<c_{-}\right\}\cup \left\{c>c_{+}\right\}$$

where

(A37) $$\left[c_{-}=\frac{{\left(\sqrt{ad}-\sqrt{be}\right)}^{2}}{f}\right]<\left[\frac{ad-be}{f}=\frac{\left(\sqrt{ad}+\sqrt{be}\right)\left(\sqrt{ad}-\sqrt{be}\right)}{f}\right]<\left[c_{+}=\frac{{\left(\sqrt{ad}+\sqrt{be}\right)}^{2}}{f}\right]$$

Therefore, according to conditions (A34) and (A36) and inequality (A37), the two positive equilibrium points exist only if:

(A38) $$0<c<c_{-}$$

Next, we assume that the condition (A38) is satisfied, and we analyze the stability of the equilibrium points:

(A39) $$\mathrm{Jacobian}\;\mathrm{Matrix}\;J=\left[\begin{array}{ccc} -g-\beta & 0 & 0\\ \beta & aI-b-2cT & aT\\ 0 & d-eI & -eT-f \end{array}\right]$$

Characteristic Equation:

(A40) $$\begin{array}{ccc} Q\left(\lambda \right) & =det\left\{\left[\begin{array}{ccc} \lambda +g+\beta & 0 & 0\\ -\beta & \lambda -aI+b+2cT & -aT\\ 0 & -d+eI & \lambda +eT+f \end{array}\right]\right\} & \\ & =\left[\lambda +g+\beta \right]\left[\lambda^{2}+\underset{U}{\underset{︸}{\left[eT+f+b-aI+2cT\right]}}\lambda +\underset{V}{\underset{︸}{\left[-af\;I+2ceT^{2}+beT+bf+2cf\;T-adT\right]}}\right] \end{array}$$

All the equilibrium points have a negative eigenvalue λ = −*g* − *β*. For the sign of other eigenvalues, the sign of coefficients of the characteristic equation has to be checked in equilibrium points. These coefficients are positive for the trivial equilibrium point and therefore, all eigenvalues have negative real value. Hence, the trivial equilibrium point is stable. For stability analysis of nontrivial equilibrium points, the sign of *U* and *V* in equation (A40) has to be analyzed:

(A41) $$U=eT+f+2cT+{\left(b-aI\right)}^{\underline{\underline{(A33):b-aI=-cT}}}\;eT+f+cT>0$$

Coefficient *U* is positive for the positive equilibrium points. Therefore, their stability depends on the sign of *V* in equation (A40):

(A42) $$\begin{array}{ll} V & =f\left(b-aI\right)+2ceT^{2}+beT+2cfT-adT^{\underline{\underline{(A33):b-aI=-cT}}}\;2ceT^{2}+\left(be-ad\right)T+cfT \end{array}$$

(A43) $$\begin{array}{ll} & =ceT^{2}+\underset{\mathrm{According}\;\mathrm{to}\;\left(A32\right):\;=0}{\underset{︸}{\left[ceT^{2}+\left(cf+be-ad\right)T+bf\right]}}-bf=ceT^{2}-bf \end{array}$$

(A44) $$\begin{array}{ll} V{|}_{T_{3}} & =\frac{1}{2ce}\left[\Delta -\underset{\mathrm{According}\;\mathrm{to}\;\left(A34\right):\;<0}{\underset{︸}{\left(cf+be-ad\right)}}\sqrt{\Delta }\right]>0 \end{array}$$

The sign of *V* for the larger equilibrium point is positive and hence, this equilibrium point is stable.

(A45) $${\left.V\right|}_{T_{2}}=\frac{\sqrt{\Delta }}{2ce}\underset{\mathrm{According}\;\mathrm{to}\;\left(A32\right):<0}{\underset{︸}{\left[\sqrt{\Delta }+\underset{\mathrm{According}\;\mathrm{to}\;\left(A34\right):\;<0}{\underset{︸}{\left(cf+be-ad\right)}}\right]}}<0$$

The sign of *V* for the smaller equilibrium point is negative and therefore, this equilibrium point is unstable. The equilibrium points of the model with *β* > 0 and by considering *βN*(*t*) = *k*(*t*) are obtained by solving:

(A46a) $$\begin{array}{ll} & -ceT^{3}-\left(cf+be-ad\right)T^{2}+\left(ek-bf\right)T+kf=0 \end{array}$$

(A46b) $$\begin{array}{ll} & I=\frac{dT}{eT+f} \end{array}$$

By keeping the assumptions (A2) and (A38), the equation (A46a) has either one positive real equilibrium point or three positive real equilibrium points depending on the value of *k*. The stability of equilibrium points that are obtained from (A46a) and (A46b) by varying the value of *k* is hard to be checked analytically; instead, it is analyzed numerically by solving equation (A40).

### A.4 Steady state analysis of model (13)

The equilibrium points of model (13) for *βN*(*t*) = *k*(*t*) = 0 can be obtained from the following

(A47) $$\begin{array}{ll} & \left(T_{1},\;I_{1},\;R_{1},\;N_{1}\right)=\left(0,\;0,\;0,\;\frac{N_{0}}{g}\right) \end{array}$$

(A48) $$\begin{array}{ll} & T_{2,3}:ceT_{2,3}^{2}+\left(cf-ad+be\right)T_{2,3}+bf=0,I_{2,3}=\frac{dT_{2,3}}{eT_{2,3}+f},R_{2,3}=0,N_{2,3}=\frac{N_{0}}{g} \end{array}$$

(A49) $$\begin{array}{ll} & T_{4}=\frac{b\left(eR_{5}+f\right)}{\epsilon ad-be},I_{4}=\frac{b}{\epsilon a},R_{4}=\left(\frac{b}{\epsilon }\right)\left(\frac{-\epsilon \left(cf+be-ad\right)+be\left(\epsilon -1\right)-\epsilon \left(\epsilon ad-be\right)}{cbe+\gamma \left(\epsilon ad-be\right)}\right),N_{4}=\frac{N_{0}}{g} \end{array}$$

(A50) $$\begin{array}{ll} & T_{5}=0,\;I_{5}=\frac{b}{\epsilon a},R_{5}=\frac{-f}{e},N_{5}=\frac{N_{0}}{g} \end{array}$$

The sign of the equilibrium point *T*_4_ changes by changing the Treg-associated parameters (ϵ, γ). *T*_4_ is positive if

(A51) $$\epsilon ad-be>0,R>-\frac{f}{e}$$

or

(A52) $$\epsilon ad-be<0,R<-\frac{f}{e}$$

The physiologically relevant regime of the model occurs by satisfying the condition (A51) which means both *T*_4_ and *R*_4_ are positive.

The equilibrium points of model (13) for *β N*(*t*) = *k*(*t*) ≠ 0 can be obtained from the following

(A53) $$\begin{array}{ll} & N=\frac{\beta N_{0}}{g+\beta } \end{array}$$

(A54) $$\begin{array}{ll} & R=\frac{-\lambda k}{\epsilon aI-b} \end{array}$$

(A55) $$\begin{array}{ll} & T=\frac{-I\left(-e\lambda k+f\epsilon aI-bf\right)}{\left(\epsilon aI-b\right)\left(-d+eI\right)} \end{array}$$

(A56) $$\begin{array}{ll} & P_{5}I^{5}+P_{4}I^{4}+P_{3}I^{3}+P_{2}I^{2}+P_{1}I+P_{0}=0 \end{array}$$

where

(A57) $$\begin{array}{ll} P_{5} & =a^{3}f\epsilon^{2}e \end{array}$$

(A58) $$\begin{array}{ll} P_{4} & =-bf\epsilon^{2}a^{2}e+cf^{2}\epsilon^{2}a^{2}-k\epsilon^{2}a^{2}e^{2}-a^{3}f\epsilon^{2}d-2\;a^{2}f\epsilon \;be-a^{2}e^{2}\lambda \;k\epsilon \end{array}$$

(A59) $$\begin{array}{llll} P_{3} & =be^{2}\lambda \;k\epsilon \;a-2\;cf^{2}\epsilon \;ab+ae^{2}\lambda \;kb+2\;a^{2}f\epsilon \;bd+\gamma \;\lambda \;kf\epsilon \;ae+ab^{2}fe+bf\epsilon^{2}a^{2}d\; & & \;\\ & \;+2\;k\epsilon^{2}a^{2}de-2\;ce\lambda \;kf\epsilon \;a+2\;b^{2}f\epsilon \;ae+a^{2}e\lambda \;k\epsilon \;d+2\;k\epsilon \;abe^{2} \end{array}$$

(A60) $$\begin{array}{llll} P_{2} & =2\;ce\lambda \;kbf-4\;k\epsilon \;abde-\gamma \;\lambda \;kbfe-b^{2}e^{2}\lambda \;k-kb^{2}e^{2}-be\lambda \;k\epsilon \;ad-b^{3}fe-ae\lambda \;kbd+cb^{2}f^{2}\; & & \;\\ & \;+ce^{2}\lambda^{2}k^{2}-ab^{2}fd-k\epsilon^{2}a^{2}d^{2}-\gamma \;\lambda^{2}k^{2}e^{2}-2\;b^{2}f\epsilon \;ad-\gamma \;\lambda \;kf\epsilon \;ad \end{array}$$

(A61) $$\begin{array}{ll} P_{1} & =\gamma \;\lambda^{2}k^{2}ed+b^{2}e\lambda \;kd+2\;kb^{2}de+2\;k\epsilon \;abd^{2}+b^{3}fd+\gamma \;\lambda \;kbfd \end{array}$$

(A62) $$\begin{array}{ll} P_{0} & =-kb^{2}d^{2} \end{array}$$

### A.5 Nonlinear proliferation rate of conventional and regulatory T cells

In the models (1), (4), (10) and (13) it is assumed that proliferation rate of Tconvs and Tregs is a linear function of IL-2. This simplifying assumption is made in order to allow parametric stability analysis of the model in a closed form and to find explicitly the dependency between parametric variations and topological changes of the model. Here, we show that the simplifying assumption does not affect the three regimes of qualitative immune responses that could be derived from the model. The linear IL-2-dependent proliferation rate is replaced with a nonlinear function of IL-2, named Φ (*I*) in models (1), (4), and (13):

(A63) $$\left\{\begin{array}{c} \frac{dT}{dt}=\Phi \left(I\right)T-bT+k\;\\ \frac{dI}{dt}=dT-eIT-fI\;\\ \; \end{array}\right.$$

where Φ (*I*) is considered as a Hill-function of IL-2

(A64) $$\Phi \left(I\right)=a\frac{I^{n}}{h^{n}+I^{n}}$$

The models (1) and (A63) are compared by steady state analysis. The equilibrium points of the modified model (A63) (with $k=\frac{\beta N_{0}}{g+\beta }=0$) are

(A65) $$\begin{array}{ll} & \left(T_{1},\;I_{1}\right)=\left(0,\;0\right) \end{array}$$

(A66) $$\begin{array}{ll} & \left(T_{2},I_{2}\right)=\left(\frac{fI_{2}}{d-eI_{2}},h{\left(\frac{b}{a-b}\right)}^{\frac{1}{n}}\right) \end{array}$$

The nontrivial equilibrium point (*T*_2_, *I*_2_) is positive and biologically meaningful only if

(A67) $$\left(a-b\right)d^{n}-be^{n}h^{n}>0$$

The local stability of the equilibrium points can be determined by obtaining the eigenvalues from the characteristic equation:

(A68) $$\begin{array}{ll} \mathrm{Characteristic}\;\mathrm{Equation}\;Q\left(\lambda \right) & =det\left\{\left[\begin{array}{cc} \lambda -a\frac{I^{n}}{h^{n}+I^{n}}+b & -aT\frac{nh^{n}I^{n-1}}{{\left(h^{n}+I^{n}\right)}^{2}}\\ -d+eI & \lambda +eT+f \end{array}\right]\right\} \end{array}$$

(A69) $$\begin{array}{llll} & =\lambda^{2}+\left[eT+f-a\frac{I^{n}}{h^{n}+I^{n}}+b\right]\lambda \; & & \;\\ & \;+\left[\left(-a\frac{I^{n}}{h^{n}+I^{n}}+b\right)\left(eT+f\right)+aT\frac{nh^{n}I^{n-1}}{{\left(h^{n}+I^{n}\right)}^{2}}\left(-d+eI\right)\right]=0 \end{array}$$

By checking Routh–Hurwitz stability Criterion (RHC) it can be easily confirmed that the eigenvalues have negative real parts for trivial equilibrium point since all the coefficients of polynomial *Q*(λ) are positive, and hence, the trivial equilibrium point (*T*_1_, *I*_1_) is locally stable. For checking the stability of the nontrivial equilibrium point, the characteristic equation (A69) is evaluated in (*T*_2_, *I*_2_):

(A70) $$\lambda_{1,2}{|}_{\left(T_{2,}I_{2}\right)}:Q(\lambda ){|}_{\left(T_{2,}I_{2}\right)}=\lambda^{2}+\underset{U}{\underset{︸}{\left[eT_{2}+f\right]}}\lambda +\underset{V}{\underset{︸}{\left[-\frac{nbh^{n}T_{2}}{I_{2}\left(h^{n}+{I_{2}}^{n}\right)}\left(d-eI_{2}\right)\right]}}=0$$

By assuming the condition (A67), the coefficients *U* and *V* are positive and negative respectively. Therefore, the sign of the coefficients of *Q*(λ) (*U* and *V*) changes only once and hence, there exists an eigenvalue with positive real part. Therefore, the nontrivial equilibrium point (*T*_2_, *I*_2_) is a saddle node and unstable.

Similar to the model (1), the stable manifold of saddle node in the model (A63) defines a threshold for the initial conditions that allow for unlimited proliferation of activated T cells. By comparing the conditions (A67) and (3), the dependencies of these conditions to the model parameters, specifically the proliferation rate (*a*) and IL-2 secretion rate (*d*), are positively correlated. In other words, in both models, only T cell clones with sufficiently high proliferation rate (*a*) and/or high IL-2 secretion rate (*d*) are able to undergo major T cell proliferation.

For *k* ≠ 0, the equilibrium points of the model (A63) are obtained from the following equations:

(A71) $$\begin{array}{ll} & T=\frac{f\;I}{d-eI} \end{array}$$

(A72) $$\begin{array}{ll} & I:\left[\left(a-b\right)f-ke\right]I^{n+1}+\left[kd\right]I^{n}-\left[h^{n}\left(bf+ke\right)\right]I+kdh^{n}=0 \end{array}$$

The stability of equilibrium points is analyzed by evaluating the characteristic equation and is shown in Figure A1 for parameter values given in Table 1 and Hill-function parameters *n* = 2 and *h* = 0.5. By comparing the bifurcation diagram in Figures A1 and 3B, the qualitative similarity between model (4) and (A63) is evident. This qualitative similarity also holds true between model (13) and the following model:

(A73) $$\left\{\begin{array}{c} \frac{dT}{dt}=\Phi \left(I\right)T-bT-cT^{2}-\gamma RT+k\;\\ \frac{dR}{dt}=\epsilon \Phi \left(I\right)R-bR+\beta \hat{N}\;\\ \frac{dI}{dt}=dT-eI\left(T+R\right)-f\;I\;\\ \; \end{array}\right.$$

where Φ (*I*) is identical to (A64).

The equilibrium points of model (A73) are calculated and their stability is analyzed by deriving the characteristic equation of the model and obtaining the eigenvalues. By keeping assumption (12), the bifurcation diagrams of model (A73) for two different values of λ are obtained by treating $k=\frac{\beta N_{0}}{g+\beta }>0$ as the bifurcation parameter (depicted in Figure A2). Depending on the value of *k*, the model has either 8 or 6 equilibrium points (4 or 2 equilibrium points with *T* > 0, identical to model (13)) with parameter values given in Table 1 and Hill-function parameters *n* = 4 and *h* = 1. The additional equilibrium points resulted from considering the Hill-function nonlinearity are all in the negative space of the model variables. As it can be seen from Figure A2, similar to the model (13), the three qualitatively different responses still could be derived from the modified model. It is clear that the value of *k_i_*, *k_s_*, and λ*_th_* are different from their corresponding values in the model (13).

In summary, imposing the nonlinear IL-2 dependent proliferation rate of cells results in a more restricted condition for initiation of an immune response in comparison to the linear IL-2 dependent proliferation rate, namely the requirement of higher T cell avidity (higher *a* and *d*), higher Ag-stimulation (increased *β_i_*), and lower Treg/Tconv ratio (lower λ_th_); but three qualitatively different immune reactions depending on the critical levels of Ag-stimulation could still be derived, very similar to model (13).
